# dbGENVOC: database of GENomic Variants of Oral Cancer, with special reference to India

**DOI:** 10.1093/database/baab034

**Published:** 2021-05-28

**Authors:** Sanchari Pradhan, Subrata Das, Animesh K Singh, Chitrarpita Das, Analabha Basu, Partha P Majumder, Nidhan K Biswas

**Affiliations:** Human Genetics Unit, National Institute of Biomedical Genomics, Kalyani, West Bengal 741251, India; Human Genetics Unit, National Institute of Biomedical Genomics, Kalyani, West Bengal 741251, India; Human Genetics Unit, National Institute of Biomedical Genomics, Kalyani, West Bengal 741251, India; Human Genetics Unit, National Institute of Biomedical Genomics, Kalyani, West Bengal 741251, India; Human Genetics Unit, National Institute of Biomedical Genomics, Kalyani, West Bengal 741251, India; Human Genetics Unit, National Institute of Biomedical Genomics, Kalyani, West Bengal 741251, India; Human Genetics Unit, Indian Statistical Institute, Kolkata, West Bengal 700108, India; Human Genetics Unit, National Institute of Biomedical Genomics, Kalyani, West Bengal 741251, India

## Abstract

Oral cancer is highly prevalent in India and is the most frequent cancer type among Indian males. It is also very common in southeast Asia. India has participated in the International Cancer Genome Consortium (ICGC) and some national initiatives to generate large-scale genomic data on oral cancer patients and analyze to identify associations and systematically catalog the associated variants. We have now created an open, web-accessible database of these variants found significantly associated with Indian oral cancer patients, with a user-friendly interface to enable easy mining. We have value added to this database by including relevant data collated from various sources on other global populations, thereby providing opportunities of comparative geographical and/or ethnic analyses. Currently, no other database of similar nature is available on oral cancer. We have developed Database of GENomic Variants of Oral Cancer, a browsable online database framework for storage, retrieval and analysis of large-scale data on genomic variants and make it freely accessible to the scientific community. Presently, the web-accessible database allows potential users to mine data on ∼24 million clinically relevant somatic and germline variants derived from exomes (*n* = 100) and whole genomes (*n* = 5) of Indian oral cancer patients; all generated by us. Variant data from The Cancer Genome Atlas and data manually curated from peer-reviewed publications were also incorporated into the database for comparative analyses. It allows users to query the database by a single gene, multiple genes, multiple variant sites, genomic region, patient ID and pathway identities.

**Database URL**: http://research.nibmg.ac.in/dbcares/dbgenvoc/

## Introduction

Oral squamous cell carcinoma (OSCC), a subset of head and neck cancer, is the 6th most common malignancy in the world (1) and one of the most prevalent cancers among males in Indian and southeast Asian population groups (2). Advancements in sequencing technologies have enabled the generation of large-scale genomic data of OSCC genomes worldwide (3–5) and from India (6, 7). These studies have cataloged both somatic and germline DNA variations, present in and specific to the tumor genomes. The need for systematic collection, unified analysis and subsequent development of a database of oral cancer genomic variants are of critical importance for research and diagnosis of this common cancer.

Current databases (Table 1) have extremely limited information on genome-scale variation of oral cancer. The Cancer Genome Atlas (TCGA) project database contains head and neck cancer (HNSCC) data (OSCC, is a subset of HNSCC) generated on patients’ resident in the USA. Similarly, International Cancer Genome Consortium (ICGC) project database contains somatic mutation data primarily on HNSCC, less so on oral cancer patients from multiple countries. None of these data resources (i) is focused on oral cancer genomic variants from India and South-east Asia and (ii) allows cross-comparison of other available datasets from a single portal.

**Table 1.** Information of data resources on cancer

Database	Description	URL	Refs
Comprehensive cancer Resources			
IARC TP53 Database	International Agency for Research on Cancer TP53 database	http://p53.iarc.fr/	(9, 10)
CGC	The Cancer Gene Census	http://cancer.sanger.ac.uk/cancergenome/projects/census/	(11)
COSMIC	Catalogue of Somatic Mutations in Cancer	http://cancer.sanger.ac.uk/cancergenome/projects/cosmic/	(12)
MethyCancer	A database of human DNA methylation and cancer	http://methycancer.psych.ac.cn/	(13)
TSGene	Tumor Suppressor Gene Database	https://bioinfo.uth.edu/TSGene/	Unpublished
Cancer Genetics Web	Cancer Genetics Web	https://www.cancer-genetics.org/	Unpublished
dbDEPC 3.0	Differentially Expressed Proteins in human Cancer	https://www.scbit.org/dbdepc3/index.php	(14–16)
Oral cancer specific databases			
HNOCDB	Head and Neck Oral Cancer Database	http://gyanxet.com/hno.html	(17)
OrCa-db	Oral Cancer Database	http://www.rgcb.res.in/orcadb	(18)
OCGB version 1	Oral Cancer Gene Database version 1	http://www.actrec.gov.in/oralcancer/GeneHome.htm	(19)
OCGB version 2	Oral Cancer Gene Database version 2	http://www.actrec.gov.in/OCDB/	(20)
OrCGDB	Oral Cancer Gene Database	http://http//www.tumor-gene.org/Oral/oral.html	(21)

There have been some earlier attempts to create population-specific database for genetic variants associated with diseases, such as Esophageal Squamous Cell Carcinoma in Chinese Population (8) and Medical Genomics Japan Variant database (https://mgend.med.kyoto-u.ac.jp/). Such population-specific, open access, genome databases of diseases do not exist in India. We have attempted to fill this gap. The overall objective of the current effort is to develop Database of GENomic Variants of Oral Cancer (dbGENVOC). dbGENVOC, is an extensive, easily explorable, open-access web portal that allows users to mine oral cancer variants (somatic and rare germline (Minor Allele Frequency (MAF) <1%) single nucleotide variants (SNVs)), and insertions and deletions identified by whole exome and whole genome sequencing from oral cancer patients drawn from India. dbGENVOC provides curated, updated and deeply annotated gene-level summary statistics for mutated genes in oral cancer. This database will be beneficial to both national and international researchers to conduct future association studies, diagnostic tests, and to perform wet-lab validation on important targets.

## Materials and methods

**Figure 1.**
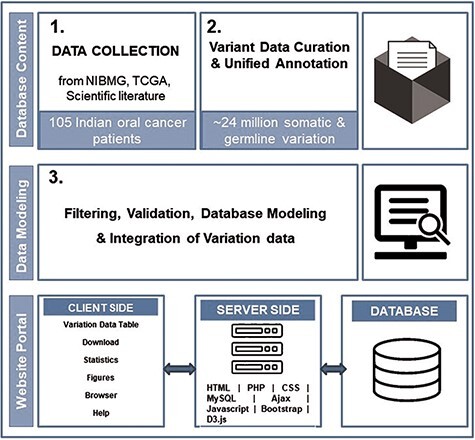
The overall schematic overview of steps to build dbGENVOC.

dbGENVOC provides a web interface for querying, visualizing and downloading individual-specific oral cancer variation data. The database has been created using open-source technologies, designed and implemented in three steps: patient data collection, variant data curation and unified annotation, database structure/web interface. Figure 1 represents the overall schematic overview of steps to build dbGENVOC.

### Data collection

The Indian oral cancer sequence BAM files for patients have been accessed from the European Genome-Phenome Archive under accession code EGAS00001000249, EGAS000010010 and EGAD00001002120. TCGA data were taken from this data freeze 10.1016/j.cels.2018.03.002, and clinical annotations were collected from cbioportal (22). After extensive screening, specific variant data on oral cancer patients were collated and annotated (5, 7, 23).

### Variant data curation and unified annotation

Followed the same protocol mentioned in our previous publication (6), Indian oral cancer data were demultiplexed and converted to FASTQ file using CASAVA (Illumina). Reads were quality controlled and filtered. Pass filtered reads were aligned to the human genome reference sequence (hs37d5) using BWA-mem (24). Best practice protocol for sorting, duplicate removal, local indel realignment and base quality score recalibration was performed using the GATK (25) package. To strengthen confidence for both somatic and germline calls, SNVs and small InDels were detected using multiple variant callers and an ensemble call set was generated for both somatic and germline variants separately. Five variant callers, i.e. Muse (26), Mutect1 (27), Mutect2 (28), Strelka2 (29) and BbB (a method developed by our team (6)) were used to generate the ensemble callset of somatic mutations. Individual variant caller specific calls were merged into a union callset. The criterion applied to include a variant in the final callset was if the variant was identified by more than one variant caller or if it was identified by only one variant caller and its read proportion was ≥0.1 with at least three variant supporting reads and at least one read with the variant in each direction.

The germline variant callset was generated from matched normal (blood) DNA sequencing data using three different variant callers (GATK-HaplotypeCaller (30), Strelka2 (29) and NIBMG-BbB caller) were obtained for ICGC-India cohort. A two-tier filter was used to make the final selection. Germline variants detected by GATK-Haplotypecaller satisfied the recommended hard filters (strand-bias, read-position, read mapping quality, quality by depth, etc.) were included in the first selected subset. From this subset, those variant sites were finally selected that satisfied one of the following criteria: (i) the genotype quality of the variant was either ≥20 and was validated by Strelka2 or NIBMG—BbBcaller variant caller or (ii) the genotype quality was ≥30.

Community recommended filters (SB, OXO-G, etc.) were applied to remove false positive variants. Freezed callsets were annotated using Oncotator (31). Variant data collected from TCGA and other scientific literature were also reprocessed and re-annotated using the Oncotator tool. Since, the germline variants of the other studies (TCGA and peer-reviewed papers) were either in embargo or not available from original source, such data could not be included in our database.

### Database structure/web interface

In the backend, the relational database is managed with MySQL. The web interface of dbGENVOC was developed using HTML, PHP, CSS, JavaScript, Ajax and libraries from Bootstrap, Datatables and JQuery. Figures accompanying the data are dynamically generated using D3.js library. The database is hosted on Ubuntu operating system run by a high-memory Apache HTTP server.

## Results

dbGENVOC can be accessed through http://research.nibmg.ac.in/dbcares/dbgenvoc/. First release of dbGENVOC currently contains data on (i) ∼24 million somatic and germline variants (20 105 539 SNVs, 1 402 298 insertions and 1 580 590 deletions) derived from whole exome sequences of 100 Indian oral cancer patient and whole genome sequences of five oral cancer patients from India, (ii) somatic variation data from 220 patient samples drawn from the USA and analyzed by TCGA-HNSCC project (3) and (iii) manually curated variation data of 118 patients from recently published peer-reviewed publications (5, 7, 23). Data on variants from non-Indian patients were incorporated into dbGENVOC for the identification of common and unique variants with Indian population-specific oral cancer data. To access germline data from dbGENVOC, a user registration is needed to avoid any ethical and data privacy issues. Table 2 shows the number of records in dbGENVOC per sequencing category.

**Table 2.** The number of records in dbGENVOC across various categories

Data source	NIBMG	TCGA	Peer-reviewed literature
Number of variants			
Exome Somatic	9336	46 860	34 026
Exome Germline	1 807 228	–	–
WGS Somatic	54 385	–	–
WGS Germline	21 217 481	–	–
Number of genes mutated			
Exome Somatic	5773	14 140	13 391
Exome Germline	16 183	–	–
WGS Somatic	8081	–	–
WGS Germline	21 143	–	–
Variation type (SNV/INS/DEL)			
Somatic mutations from exome data			
SNV	9092	45 193	27 908
Insertion	58	481	2215
Deletion	186	1184	3903
Germline mutations from exome data			
SNV	1 781 097	–	–
Insertion	11 031	–	–
Deletion	15 100	–	–
Somatic mutations from WGS Data			
SNV	50 563	–	–
Insertion	1299	–	–
Deletion	2523	–	–
Germline mutations from WGS data			
SNV	18 264 790	–	–
Insertion	1 389 910	–	–
Deletion	1 562 781	–	–

### dbGENVOC search options

Current features include search options by (i) Gene name: a search can be performed by any gene symbol or gene alias of a particular gene (e.g. *BRCA2*). A live search box is implemented to check whether a user has entered a proper human gene name.

(ii) Genomic region: on the basis of genomic region (e.g. 1:915 188–1 956 479) a search can be performed to get specific details of the mutation spectrum on a particular chromosomal region. For timely return of results, query region size is restricted to <100 kb.

(iii) Multi-gene: multiple genes (e.g. *TTN BRCA2 PANK4*) can be searched in a single query.

(iv) Multi-sites: multiple nucleotide positions (e.g. chr11:534 289, chr17:7 578 406 and chr17:7 577 538) search is implemented. Multiple hotspot mutations search on a gene is available.

(v) Patient ID: patient ID-specific search (e.g. NIBMG-S501-GB) was implemented.

(vi) Pathway-based search: search can be done using pathway name. An autosuggest search box is also provided for pathway-based search (e.g. Wnt signaling pathway).

(vii) Filtering utility within searched result:

A second level of query options has been incorporated in the ‘result page’. Users can further filter the result data using any additional search term, e.g. genome change, codon change, Single Nucleotide Polymorphism (SNP), variant class, variant type wise, etc. Sorting and downloading options are also provided to download the search results in an excel file.

### Database web implementation

**Figure 2.**
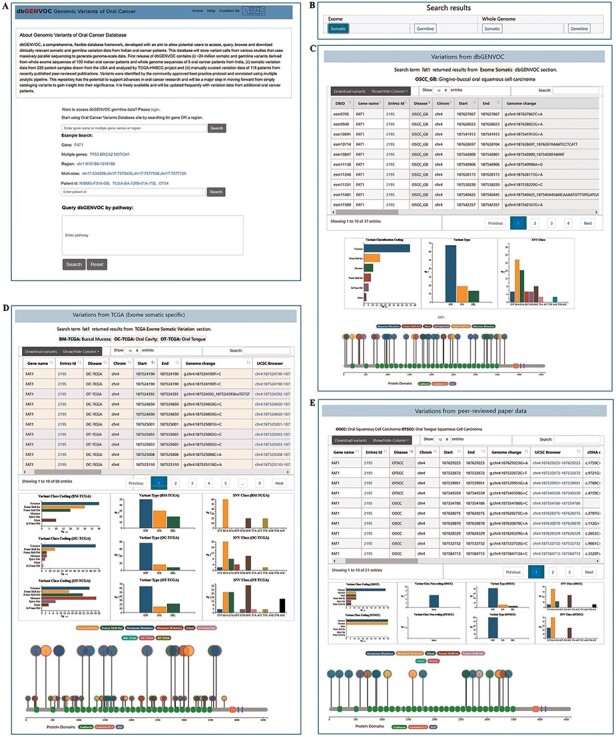
Screenshot of home page and result page. (A) Search for variants by single gene, multi-genes, region, multi-sites, patient ID and pathway identifier. (B) Search result can be explicated by ‘exome somatic’, ‘exome germline’, ‘whole genome somatic’ and ‘whole genome germline’ variant data by clicking the respective buttons. (C) Search result variants information and corresponding graphical representation from Indian oral cancer patients. (D) Search result variants information and corresponding graphical representation from TCGA-HNSCC data. (E) Search result variants information and corresponding graphical representation from scientific literature oral cancer patients’ data.

To access the generated dbGENVOC, there are mainly two web pages. The first page (Home page) includes all these search options: single gene, multiple genes, multiple variant sites, genomic region, patient ID and pathway identities. At the above panel of home page (Figure 2A), there are three pages’ link ‘home’, ‘help’, ‘contact Us’. In the middle of the home page, there are three live search boxes through which user can enter (i) either single or multiple genes, multi-sites and region, (ii) patient ID and (iii) pathway name and clicking the ‘search’ button can navigate to the ‘result page’. In the second page (result page), to show data from Indian (Figure 2C), TCGA-HNSCC (Figure 2D) and scientific literature (Figure 2E), there are three separate panels which provides individual-specific details variant information list (nucleotide change, amino acid change due to mutation, reference allele, tumor allele, chromosome, gene, sample id, annotation transcript, transcript exon, transcript position, data collection source information, etc.) including graphical representation based on variant class, variant type, SNV classification (substitution type). The variant list can be filtered, searched and exported. Lollipop diagram has been included to visualize amino acid changes or recurrent mutations with protein structure, retrieved from the stored UniProt (https://www.uniprot.org/) database. Based on search term user can also explicit ‘exome somatic’, ‘exome germline’, ‘whole genome somatic’, ‘whole genome germline’ variant data by clicking the respective buttons provided on the above section of the result page (Figure 2B). For better user convenience, a documentation page (help page) is also introduced. Additionally, a detailed contact list is provided to welcome any suggestions or query related to database.

## Utility

Data availability facilitates gene and pathway-centric mutation-specific analysis. e.g. *TP53, HRAS* and *PIK3CA* hotspot mutation search (Supplementary Figures S1–S3), mutation spectrum exploration in P53 signaling pathway, WNT signaling pathway, (Supplementary Figures S4 and S5), hotspot mutation finding in selective oncogenes (Supplementary Figure S6) and tumor suppressor genes (Supplementary Figure S7). Most frequent oral cancer gene mutations in Indian population, three different subtypes of TCGA-HNSCC (oral tongue, oral cavity and buccal mucosa) and data from other peer-reviewed publications will be automatically viewed from lollipop plot on dbGENVOC data in the same search query.

## Conclusion and future direction

To the best of our knowledge, dbGENVOC is the most comprehensive population-specific large-scale open-access database of oral cancer genomic variations. It can provide valuable insights to researchers on large-scale cancer genomic data at both population and at the individual patient level. This use of such a data repository cannot be completely enumerated. We believe that dbGENVOC will help researchers to mine important information on population-specific cancer associated variants that may also be of functional significance and may help in defining sub-phenotypes of oral cancer. dbGENVOC will be updated annually with variation data from new oral cancer patients from different regions of India and southeast Asia.

## Supplementary Material

baab034_Supp
