# Supplementary material for: dbGENVOC: database of GENomic Variants of Oral Cancer, with special reference to India
Source: Database (Oxford). 2021 May 28;2021:baab034. doi: 10.1093/database/baab034 (PMC8163239; doi:10.1093/database/baab034)
Supplement: baab034_Supp [file baab034_supp.zip › Supplementary Information.docx]

**SUPPLEMENTARY INFORMATION**

Supplementary Table 1: Peer-reviewed papers from which data were manually collated, curated and included in dbGENVOC, after screening and excluding several hundred relevant publications for lack of key data, such as chromosome number, start and/or end nucleotide positions, reference allele and/or alternate allele nucleotide identity.

| **Pubmed Id** | **Title** | **Year** | **Data Collected** |
| --- | --- | --- | --- |
| 26834999 | Integrated Analysis of Oral Tongue Squamous Cell Carcinoma Identifies Key Variants and Pathways Linked to Risk Habits, HPV, Clinical Parameters and Tumor Recurrence | 2015 | Yes |
| 28939077 | Genomic characterization of tobacco/nut chewing HPV-negative early stage tongue tumors identify *MMP10* as a candidate to predict metastases | 2017 | Yes |
| 28878238 | APOBEC3A Is an Oral Cancer Prognostic Biomarker in Taiwanese Carriers of an APOBEC Deletion Polymorphism | 2017 | Yes |


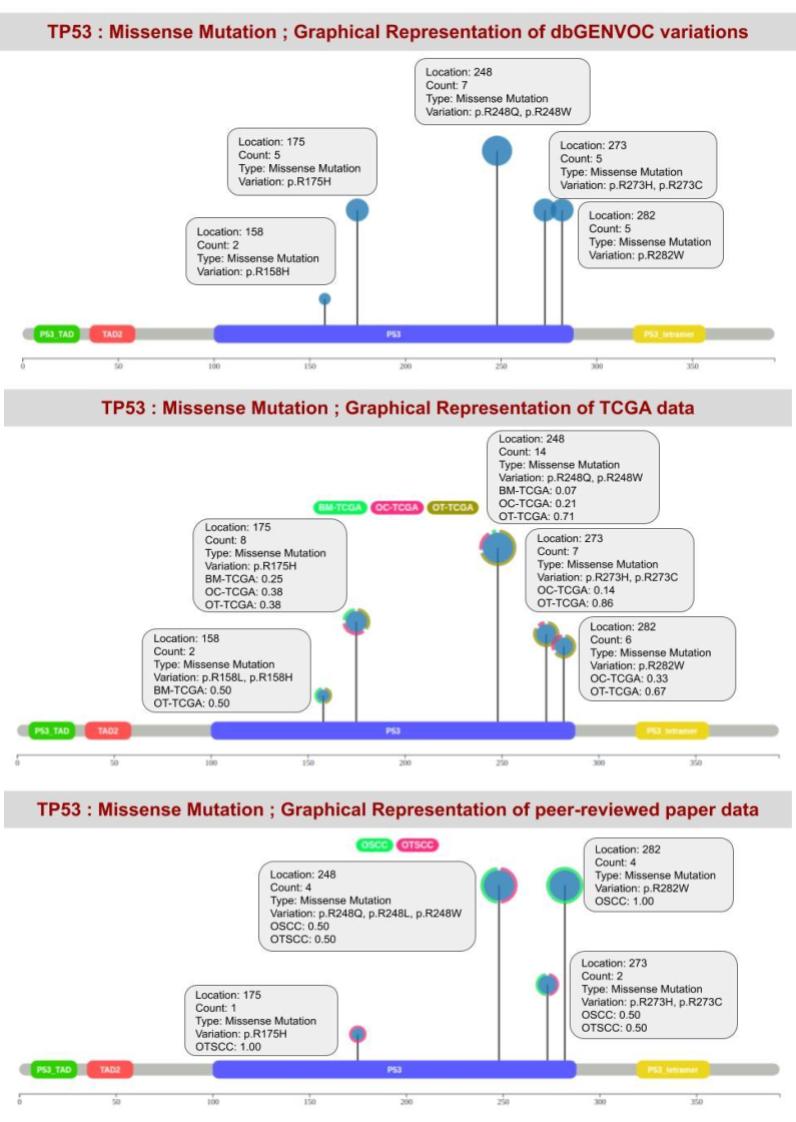


Supplementary Figures S1: dbGENVOC search result for TP53 hotspot positions provide a comparative view from three different sets of somatic exome data. The upper lollipop plot represents hotspot variation status in Indian data, middle plot represents hotspot variation status in TCGA-HNSCC data and the lower one represents hotspot variation status in data collected from peer-reviewed publications.


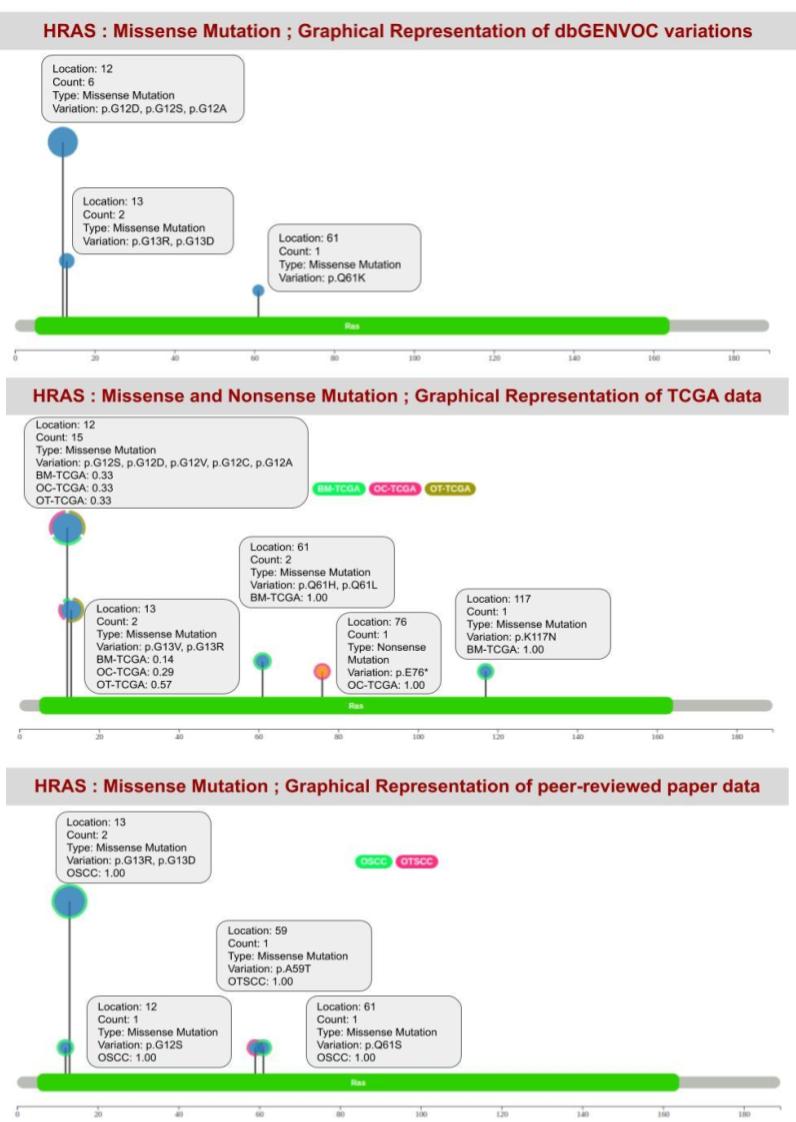

Supplementary Figures S2: dbGENVOC search result for HRAS hotspot positions provide a comparative view from three different sets of somatic exome data. The upper lollipop plot represents hotspot variation status in Indian data, middle plot represents hotspot variation status in TCGA-HNSCC data and the lower one represents hotspot variation status in data collected from peer-reviewed publications.


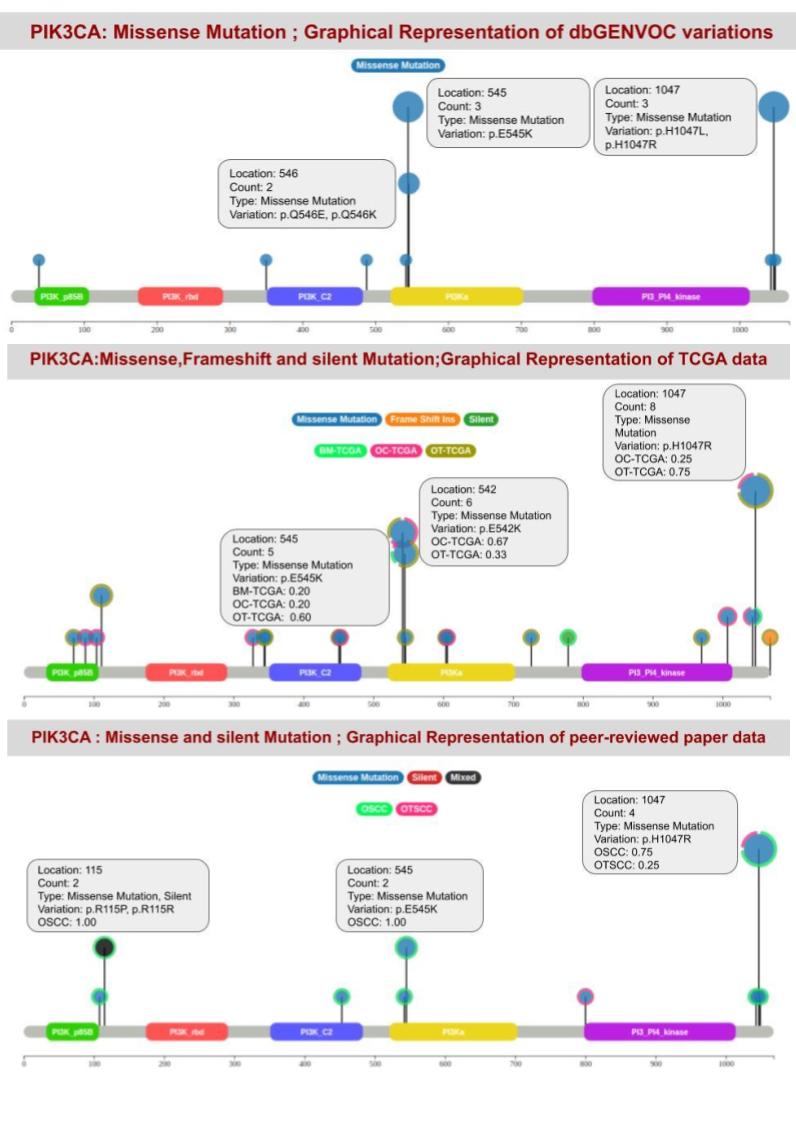


Supplementary Figures S3: dbGENVOC search result for PIK3CA variations provide a comparative view from three different sets of somatic exome data. The upper lollipop plot represents variation status in Indian data, middle plot represents variation status in TCGA-HNSCC data and the lower one represents variation status in data collected from peer-reviewed publications.


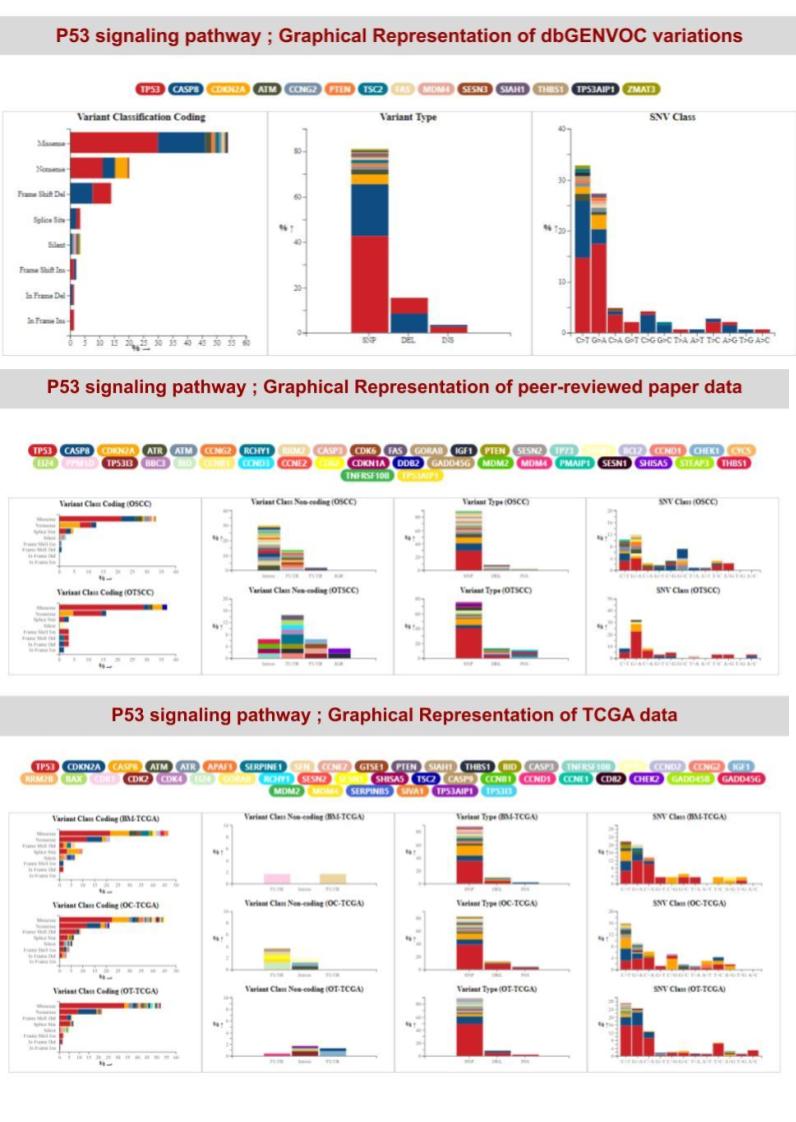


Supplementary Figures S4: dbGENVOC search results for P53 signaling pathway provide a comparative view from three different sets of somatic exome data; i) Indian data, ii) data collected from peer-reviewed publications iii) TCGA-HNSCC data. From each dataset different frequency graphs were generated based on variant class, variant type and SNV classification (substitution type) for coding, noncoding and different tumor subtypes.


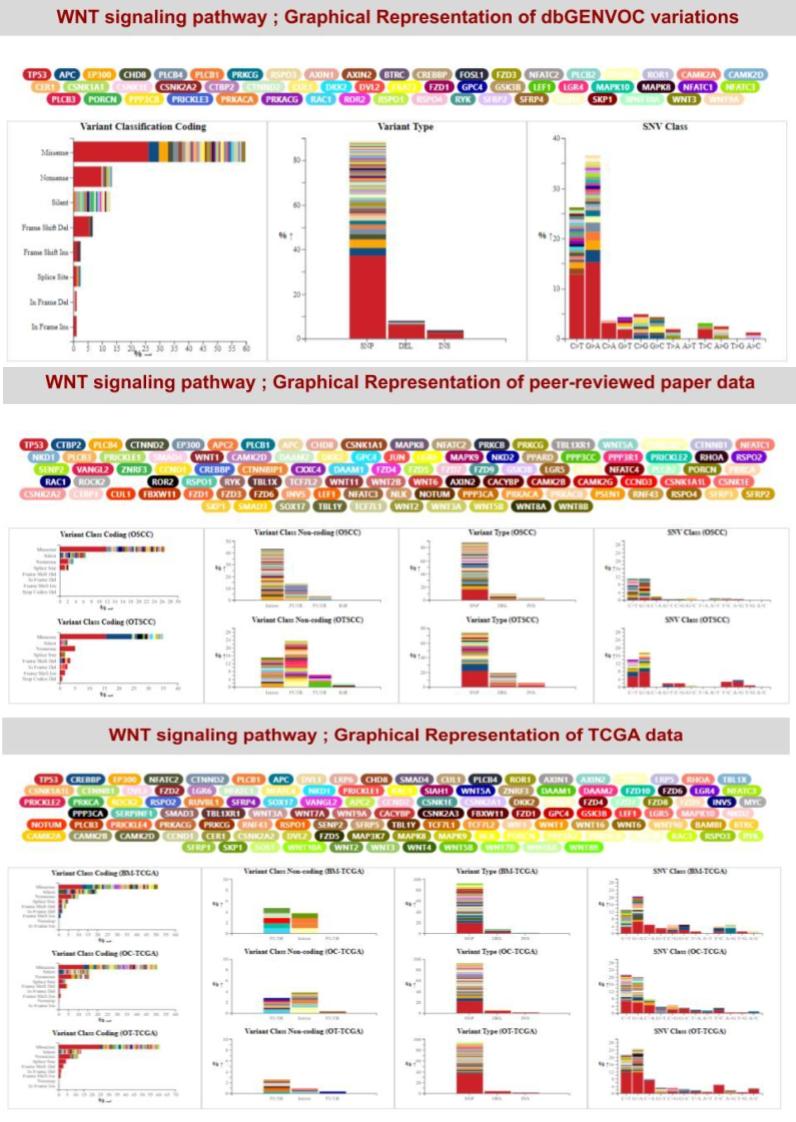


Supplementary Figures S5: dbGENVOC search results for WNT signaling pathway provide a comparative view from three different sets of somatic exome data; i) Indian data, ii) data collected from peer-reviewed publications iii) TCGA-HNSCC data. From each dataset different frequency graphs were generated based on variant class, variant type and SNV classification (substitution type) for coding, noncoding and different tumor subtypes.


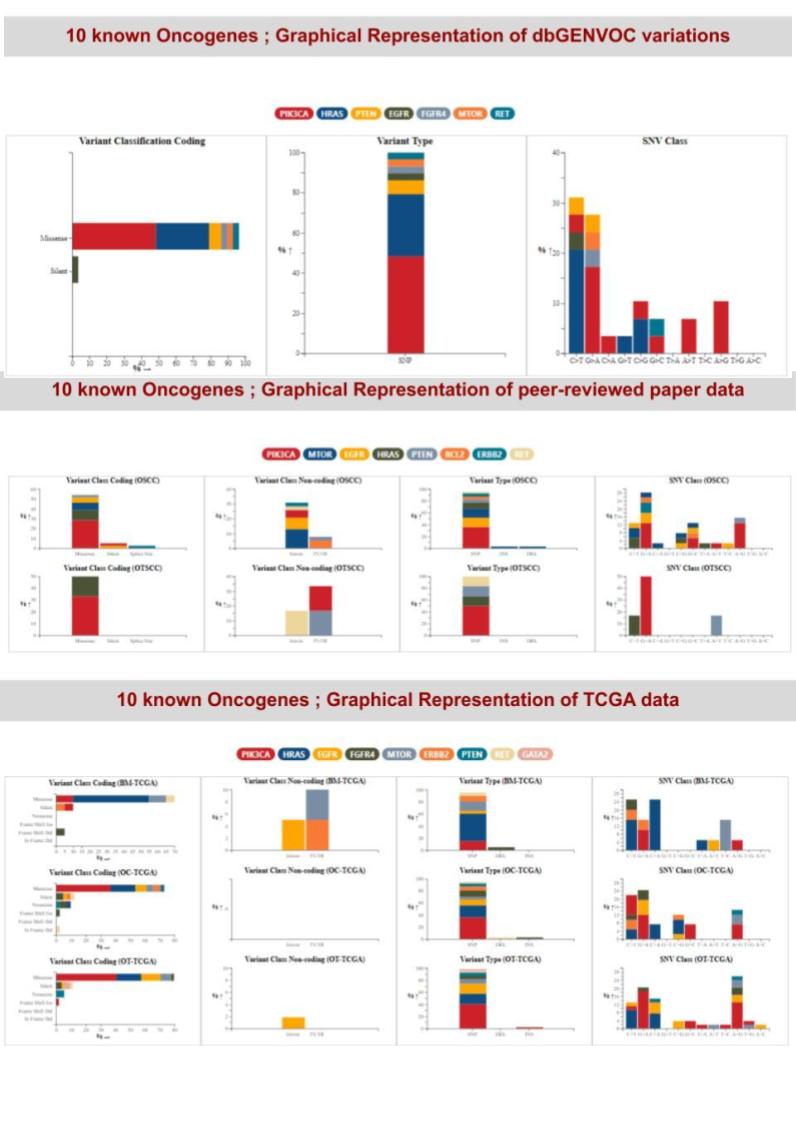


Supplementary Figures S6: dbGENVOC search results for 10 known Oncogenes (HRAS, PIK3CA, RET, FGFR4, EGFR, PTEN, ERBB2, BCL2, GATA2, MTOR) provide a comparative view from three different sets of somatic exome data; i) Indian data, ii) data collected from peer-reviewed publications iii) TCGA-HNSCC data. From each dataset different frequency graphs were generated based on variant class, variant type and SNV classification (substitution type) for coding, noncoding and different tumor subtypes.


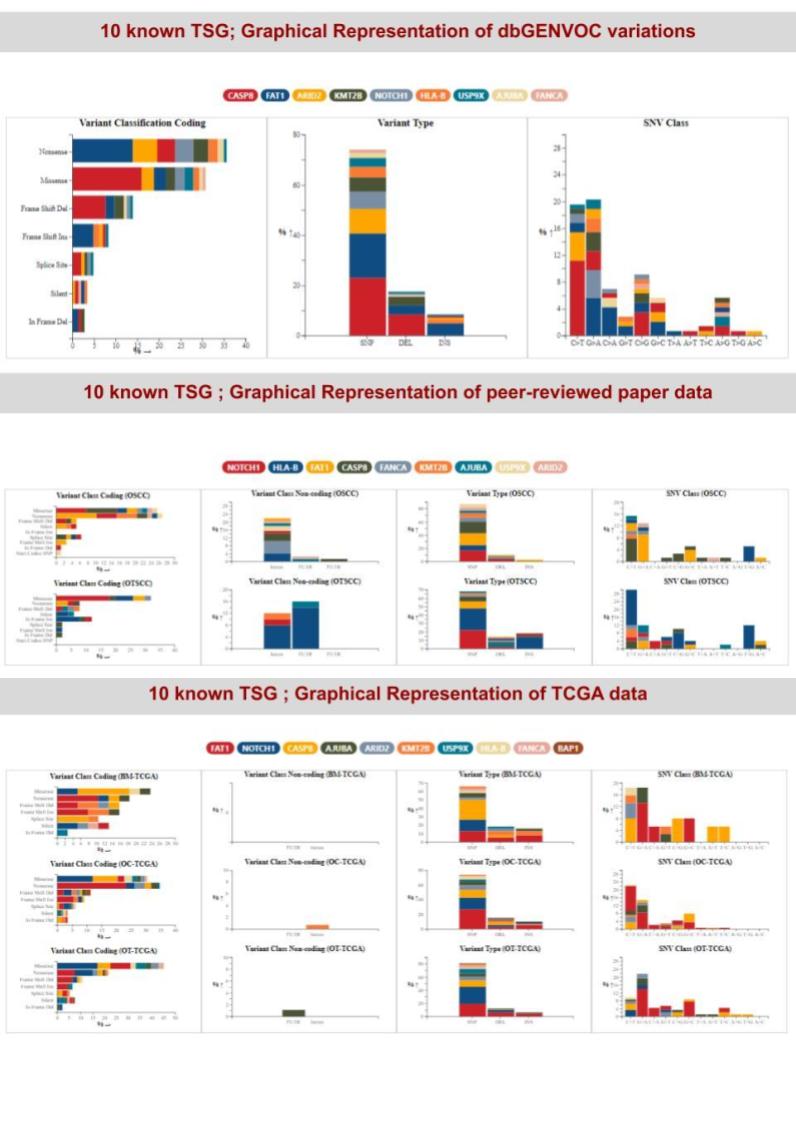


Supplementary Figures S7: dbGENVOC search results for 10 known Tumor suppressor genes (TSG) (USP9X, KMT2B, NOTCH1, CASP8, ARID2, HLA-B, AJUBA, FAT1, BAP1, FANCA) provide a comparative view from three different sets of somatic exome data; i) Indian data, ii) data collected from peer-reviewed publications iii) TCGA-HNSCC data. From each dataset different frequency graphs were generated based on variant class, variant type and SNV classification (substitution type) for coding, noncoding and different tumor subtypes.
